# Compulsory and recommended vaccination in Italy: evaluation of coverage and non-compliance between 1998-2002 in Northern Italy

**DOI:** 10.1186/1471-2458-5-42

**Published:** 2005-04-21

**Authors:** Serena Stampi, Rita Ricci, Isa Ruffilli, Franca Zanetti

**Affiliations:** 1 Department of Medicine and Public Health, Division of Hygiene, University of Bologna, Via San Giacomo 12, Bologna, Italy; 2 Bologna Health Unit, Community Pediatric Service, Bologna East District, Via Zanolini, 2, Bologna, Italy

## Abstract

**Background:**

Since vaccinations are an effective prevention tool for maintaining the health of society, the monitoring of immunization coverage allows us to identify areas where disease outbreaks are likely to occur, and possibly assist us in predicting future outbreaks. The aim of this study is the investigation of the coverage achieved for compulsory (diphtheria, tetanus, polio, hepatitis B,) and recommended (pertussis, Haemophilus influenzae, measles-mumps-rubella) vaccinations between 1998 and 2002 in the municipality of Bologna and the identification of the subjects not complying with compulsory and recommended vaccinations.

**Methods:**

The statistics regarding vaccinal coverage were elaborated from the data supplied by the Bologna vaccinal registration system (1998–2000) and the IPV4 program (2001–2002). To calculate the coverage for compulsory vaccinations and cases of non-compliance reference was made to the protocol drawn up by the Emilia Romagna Regional Administration. The reasons for non-compliance were divided into various categories

**Results:**

In Bologna the levels of immunization for the four compulsory vaccinations are satisfactory: over 95% children completed the vaccinal cycle, receiving the booster for anti-polio foreseen in their 3^rd ^year and for anti-dyphteria, tetanus, pertussis at 6 years. The frequency of subjects with total non-compliance (i.e. those who have not begun any compulsory vaccinations by the age of one year) is generally higher in Bologna than in the region, with a slight increase in 2002 (2.52% and 1.06% in the city and the region respectively). The frequency of the anti-measles vaccination is higher than that of mumps and rubella, which means that the single vaccine, as opposed to the combined MMR (measles-mumps-rubella) was still being used in the period in question.

The most common reason for non compliance is objection of parents and is probably due to reduction of certain diseases or anxiety about the possible risks.

**Conclusion:**

In Bologna the frequency of children aged 12 and 24 months who have achieved compulsory vaccination varied, in 2002, between 95% and 98%. As regards recommended vaccinations the percentage of coverage against Haemophilus influenzae is 93.3%, while the levels for measles, mumps and pertussis range from 84% to approx. 92%. Although these percentages are higher if compared to those obtained by other Italian regions, every effort should be made to strengthen the aspects that lead to a successful vaccinal strategy.

## Background

Vaccinations are an effective prevention tool for maintaining the health of the individual and society as a whole. In Italy, however, certain diseases that are preventable with vaccines whose efficiency and safety have been fully tested and proven, still present a public health issue.

Monitoring immunization coverage allows us to identify areas where disease outbreaks are likely to occur, and possibly assist us in predicting future outbreaks. Also, monitoring can help keep immunization coverage high. One of the main aims of public health services throughout the world is therefore to obtain reliable data about the level of immunization with a view to supporting the implementation and monitoring of vaccination programs [1].

Vaccinal coverage is normally referred to in terms of the percentage of subjects vaccinated out of the total target population. In pediatric age the target population is made up of the cohorts of children who were under one year old as of 31 December in a given locality, while the vaccinal state is defined as the number of doses required to bring about immunity. To determine vaccinal coverage, the WHO (World Health Organization), on a yearly basis, considers the percentage of children who have received three doses of vaccines against diphtheria, tetanus, polio, hepatitis B, pertussis and Haemophilus influenzae (Hib), and one dose of anti-measles, mumps and rubella (MMR) by the age of 24 months.

In Italy the vaccination coverage of newborns (i.e. the relationship between the number of doses of vaccine administered at a certain age in a given geographical area and the resident population of the same age) is estimated every year by the regional authorities. The data are then sent to the Ministry of Health. In order to have a more detailed picture of the national situation (to identify areas lacking coverage, to confirm coverage where present and to obtain further details, for example, about reasons for delay in application), a cluster survey, called ICONA, was made in 1998 by means of interviews carried out at the subjects' home. The results showed a considerable difference between the coverage achieved for compulsory (diphtheria, tetanus, polio, hepatitis B) and for recommended (pertussis, Hib, measles, mumps ands rubella) vaccines [2].

After five years the national survey was repeated (ICONA 2003) [3], with the particular aim of checking the data supplied by regional authorities to the Ministry and investigating the reasons for non-compliance. The study revealed that while the situation had improved, there were still differences between one region and another.

In the wake of the approval of the National Health Plan (NHP) to eliminate measles and congenital Rubella (13 November 2003) and considering the current attempt to overcome the existing division between compulsory and recommended vaccinations, we decided to undertake a study with the following objectives:

1. To investigate the coverage achieved for compulsory and recommended vaccinations between 1998 (the year when the NHP set the objective of 95% coverage) [4] and 2002 in the municipality of Bologna (capital of Emilia-Romagna region), which coincides with the East and West Districts of the city's health service;

2. To identify the categories of subjects not complying with compulsory and recommended vaccinations

## Methods

In Bologna the vaccination of subjects under 18 years of age is carried out almost exclusively by the Community Pediatric Service (which combines the former pediatric surgeries and the Nursery and Infant School Medical Service). In the current make-up of Bologna Health Services, this operative Unit is situated within the Department of Primary Care, and is divided into two districts: Bologna East and Bologna West.

Until 2000 the registration of vaccinations was carried out by the Public Health Department by means of a computerized system run by the Municipality of Bologna. The office had access to registration records (they could check the issuing of vaccination certificates), but not to the more detailed data processing system (search for cases of non-compliance, new residents etc).

Details of births or new residents under the age of 6 years were passed on to the subject's local pediatric consultation centre by the municipal Registry Office, with the issuing of a vaccination booklet. At the end of the year the Office printed a list of non-compliances and sent it to the respective Pediatric Centres for a further check. Finally, full details were sent yearly to the Regional offices by the Community Pediatric Service. During the year health workers could check the extent of vaccinal coverage only by means of the paper documents (files) sent at the same time as the vaccination booklets. The only way that the employees who registered the vaccinations on the files could find out about subjects who had moved or died was by going directly to the municipal registry office.

Since the beginning of 2001 the registration of vaccinations has been run exclusively by the Local Health Services of the city of Bologna, using a special computer program (called IPV4), *"for the purposes of the management of vaccinal registration, the municipality periodically transmits details of population variations (births, immigrations, emigrations, deaths) to the Local Health Offices" *(art. 160, paragraph 3 of the Municipal Hygiene Regulations).

Alongside the installation of the vaccinal registration program, a project was launched to set up an on-line link between the various centres administrating the vaccines at pediatric age. This allows the immediate registration of vaccinal data at the moment of vaccination and the possibility to consult in real time the vaccinal situation of each subject, including details of any migration and access to the so-called "notes" (possible contra-indications, previous adverse reactions, refusals etc).

The great advantage of the revised system is that it is now possible to monitor vaccinal coverage at any time, obtain information pertaining to moves or deaths, and contact residents who have not been vaccinated.

The statistics regarding vaccinal coverage were elaborated from the data supplied by the vaccinal registration system of the Municipality of Bologna (1998–2000) and the IPV4 program (2001–2002).

To calculate the coverage for compulsory vaccinations and cases of non-compliance reference was made to the protocol drawn up by the Emilia Romagna Regional Administration:

### compulsory vaccinations at 12 months

residents who reached one year during the year of reference and received at least 2 doses of each compulsory vaccine by the age of 12 months;

### compulsory vaccinations at 24 months

residents who reached two years during the year of reference and received at least 3 doses of each compulsory vaccine by the age of 24 months.

### subjects totally or partially non-complying

subjects who did not receive any compulsory vaccination by the age of one year or received only one dose of the compulsory vaccinations.

The reasons for non-compliance are categorized matching those used in the collection of regional data for coverage:

- living abroad: children resident in Bologna but living abroad for a long period

- refusals or objectors: children whose parents were explicitly against at least one compulsory vaccination

- immigrants: children from other regions of Italy or from other countries

- delays: children who received at least one vaccination after the pre-established dates

- other: children with health contra-indications, momentarily exempt for health reasons, not traceable or without fixed abode.

For all recommended vaccinations (anti pertussis, anti Hib, anti measles-mumps-rubella) the extent of coverage was calculated at 24 months and also at 13 years for anti-measles and anti-rubella.

## Results

The levels of immunization for the four compulsory vaccinations (taken as a whole) achieved during the five-year period in question are shown in table 1. In the municipality of Bologna these levels are satisfactory (>95%), but are lower than the overall levels reached in Emilia Romagna, although from the year 2000 the percentage of children vaccinated in the region also shows a slight decrease. Interestingly, the statistics differ from one district of the city to another: in Bologna East non-compliance tends to be higher.

**Table 1:** Levels of vaccinal coverage at 12 months (2 doses) and 24 months (3 doses) for compulsory vaccinations in the municipality of Bologna and the Emilia Romagna region

		12 months		24 months	
			
		resident population		resident population	
		N	%	N	%
1998					
	Municipality of Bologna	2561	97.11	2398	94.33
	Emilia Romagna	30585	98.70	29662	98.40
1999					
	Municipality of Bologna	2464	96.15	2560	95.90
	Emilia Romagna	31567	98.41	30916	98.30
2000					
	Municipality of Bologna	2630	97.30	2429	96.62
	Emilia Romagna	32423	98.40	31727	98.10
2001					
	Municipality of Bologna	2755	97.10	2619	96.33
	Emilia Romagna	34728	98.05	32866	98.01
2002					
	Municipality of Bologna	2741	95.81	2706	96.01
	Emilia Romagna	34969	97.64	34950	97.45

Data for the assessment of each separate vaccination are available only from 2001 (table 2). There is a clear trend towards a decline in the percentage of coverage in Bologna, which becomes even more evident in 2002. There are also differences between the compulsory vaccinations: anti-DT has the highest coverage, anti-polio intermediate and anti-hepatitis B the lowest. Regarding the vaccination against Hepatitis B, the percentage of subjects in Bologna who had completed the cycle by the age of 13 years passed from 95.0% in 1988 to 96.4% in 2002. In the same period the mean national levels of coverage were more than one percent lower, with an opposite trend to that of the Emilia Romagna region.

**Table 2:** Levels of vaccinal coverage at 12 months (2 doses) and 24 months (3 doses) for compulsory vaccinations in the municipality of Bologna and the Emilia-Romagna region

		12 months			24 months		
			
		DPT	Polio	Hepatitis B	DPT	Polio	Hepatitis B
		%	%	%	%	%	%
2001							
	Municipality of Bologna	98.7	98.5	97.1	98.3	97.9	96.5
	Emilia Romagna	98.5	98.4	98.1	98.5	98.4	98.0
	Italy				96.1	96.0	94.7
2002							
	Municipality of Bologna	96.7	96.7	95.9	97.5	97.4	96.0
	Emilia Romagna	98.2	98.2	97.7	98.1	98.0	97.5
	Italy*				96.9	96.7	95.7

* estimated data at 31 October 2003

In the city of Bologna, as in the region taken as a whole, over 95% children completed the vaccinal cycle, receiving the booster for anti-polio foreseen in their 3^rd ^year and for anti-DPT at 6 years. Once again there is a marked difference between the two Districts considered (Polio: East *vs *West = 95.8% *vs *98.3%; DPT East *vs *West = 93.45% *vs *96.8%).

The frequency of subjects with total non-compliance (i.e. those who have not begun any compulsory vaccinations by the age of one year) is generally higher in Bologna (especially in the East District) than in the region, with a slight increase in 2002 (2.52% and 1.06% in the city and the region respectively) (table 3).

**Table 3:** Frequency of subjects with total non-compliance (*) for compulsory vaccinations in the municipality of Bologna (Bologna East and Bologna West), and in the Emilia Romagna region

	1998	1999	2000	2001	2002
	%	%	%	%	%
Bologna East	0.65	1.74	1.14	0.89	2.67
Bologna West	1.12	0.17	0.76	0.36	2.34
Municipality of Bologna	0.88	0.97	0.95	0.62	2.52
Emilia Romagna	0.40	0.62	0.52	0.78	1.06

(*) subjects that have not started any compulsory vaccination by the age of one year

The vaccination registration system provides us with information about the reasons for non-compliance for the compulsory vaccinations: some can be attributed to the conditions of the children (postponements or exemptions for medical reasons, living abroad), others to the parents (untraceable, objectors-refusals). At 12 months the most common reason for non-compliance is "parents who object to and refuse the vaccinations" (1.01–1.56% of children resident in Bologna) (table 4). At 24 months the reasons vary more from year to year (table 5). In 2001 and 2002 the frequency of parents who stated they were against the anti-hepatitis B vaccination (both at 12 months and 24 months) was higher than that of the other compulsory vaccinations.

**Table 4:** Frequency of non-compliance for compulsory vaccinations at 12 months (2 doses) divided by category

		resident population	living abroad	no fixed abode	refusals objectors	immigrated	delays	other*
		N.	%	%	%	%	%	%
1998								
	Municipality of Bologna	2561	0.59	0.08	1.01	0.23	0.66	0.31
	Emilia Romagna	30585	0.22	0.06	0.35	0.18	0.27	0.13
1999								
	Municipality of Bologna	2464	0.93	0.24	1.30	0.49	0.28	0.61
	Emilia Romagna	31567	0.32	0.07	0.57	0.22	0.30	0.11
2000								
	Municipality of Bologna	2630	0.00	0.11	1.37	0.61	0.42	0.19
	Emilia Romagna	32423	0.21	0.05	0.22	0.57	0.37	0.19
2001								
	Municipality of Bologna	2755	0.11	0.04	1.56	0.22	0.54	0.43
	Emilia Romagna	34728	0.20	0.02	0.91	0.20	0.39	0.22
2002								
	Municipality of Bologna	2741	0.62	0.11	1.39	0.91	0.33	0.84
	Emilia Romagna	34969	0.21	0.06	1.06	0.24	0.42	0.37

* postponed or exempted for medical reasons, untraceable, etc.

**Table 5:** Frequency of non-compliance for compulsory vaccinations at 24 months (3 doses) divided by category

		resident population	living abroad	no fixed abode	refusals objectors	immigrated	delays	other*
		N.	%	%	%	%	%	%
1998								
	Municipality of Bologna	2398	0.71	0.12	1.00	0.25	2.04	1.54
	Emilia Romagna	29662	0.24	0.09	0.29	0.22	0.51	0.22
1999								
	Municipality of Bologna	2560	1.09	0.43	0.47	0.78	1.05	0.27
	Emilia Romagna	30916	0.27	0.07	0.35	0.22	0.37	0.11
2000								
	Municipality of Bologna	2429	0,00	0.08	0.54	1.19	0.86	0.70
	Emilia Romagna	31727	0.20	0.00	0.20	0.40	0.50	0.2
2001								
	Municipality of Bologna	2619	0.04	0.19	1.26	0.38	1.07	0.53
	Emilia Romagna	32866	0.25	0.05	0.60	0.26	0.58	0.25
2002								
	Municipality of Bologna	2706	0.00	0.04	1.55	0.59	1.07	0.74
	Emilia Romagna	34950	0.21	0.04	1.00	0.23	0.69	0.38

* postponed or exempted for medical reasons, untraceable,

Data regarding the coverage at 24 months for the recommended vaccinations confirm the decline in the vaccination of children in Bologna as compared to the rest of the region (table 6), a trend already noted for the compulsory vaccinations.

**Table 6:** Levels of vaccinal coverage at 24 months and 13 years for recommended vaccinations in the municipality of Bologna (BO) and the Emilia-Romagna region (ER)

	1998		1999		2000		2001		2002	
					
	BO	ER	BO	ER	BO	ER	BO	ER	BO	ER
	%	%	%	%	%	%	%	%	%	%
24 months										
vaccine against										
pertussis	95.7	96.8	94.2	96.1	95.1	96.7	94.9	96.8	94.8	96.8
*H. influenzae*	41.8	50.6	45.0	66.8	65.9	81.0	86.9	90.6	93.3	95.2
measles	79.3	88.2	82.4	89.1	83.5	90.4	86.4	90.7	90.2	92.3
mumps	-	-	78.8	84.2	83.3	89.4	85.1	90.1	88.8	91.7
rubella	77.2*	85.2*	78.6	88.0	83.4	89.4	85.6	90.1	88.8	91.7
										
13 years										
vaccine against										
measles	75.1	83.8	72.5	89.1	85.8	84.9	90.1	87.4	92.6	91.3
rubella	84.7*	85.2*	49.9	60.8	72.6	66.3	76.1	73.4	84.2	80.6

* data available for females only

The underlying trend for such coverage tends to vary over the five-year period. The level of protection against pertussis remains more or less constant, even though it falls a little below the regional average. However, a steady rise can be seen for the other recommended vaccinations, especially evident in the case of anti-Hib (41.8% and 50.6% of children resident in Bologna and Emilia Romagna respectively in 1998, against 93.3% and 95.2% in 2002). The notable increase in anti-Hib vaccination can probably be attributed to the availability of a combined vaccine.

At 12 months a similar consistent increase in the number of subjects receiving the anti-Hib vaccination can again be seen in Bologna (from 26.7% to 91.6%) and throughout the whole of the region (from 40.9% to 96.0%). In Bologna the target percentage of 95% coverage for the recommended vaccinations has yet to be reached.

Table 6 also shows how the frequency of the anti-measles vaccination is higher than that of mumps and rubella, which means that the single vaccine, as opposed to the combined MMR, was still being used in the period in question. From the year 2000, the levels of coverage reached in Bologna for anti-measles and anti-rubella at 13 years of age would seem to indicate a greater attention to these vaccinations in Bologna than in other parts of the region. Unlike the findings for the compulsory and recommended vaccinations, no difference was found between the two Districts of the city.

## Discussion

The principal infant vaccinations have now been included in the Essential Levels of Assistance that the Region must guarantee free of charge to all citizens. Despite this, however, the levels of coverage for compulsory and recommended vaccinations have not yet reached homogeneous levels. In the municipality of Bologna the frequency of children aged 12 and 24 months who have been vaccinated against DTP, tetanus, polio, hepatitis B and pertussis varied, in 2002, between 95% and 98%. As far as the recommended vaccinations are concerned, the percentage of coverage against Hib is 93.3%, while the levels for measles, mumps and pertussis range from 84% to approx. 92%. Although these percentages are generally lower than the regional levels, they can nevertheless be considered fairly good if compared to those obtained by certain other Italian regions [5] and Italy as a whole. The results achieved at a national level for vaccinations against polio, DTP and hepatitis B were respectively 96.6%, 95.3% and 94.1% in 2000 and 96.7%, 96.9% and 95.7% in 2002 [3]. Moreover, the anti-Hib vaccination was given to 54.7%, 71.2% and 84.4% of children in Italy respectively in the years 2000, 2001 and 2002, while the mean coverage for MMR was 74.1,%, 76.5% and 81.1% (Ministry of Health). In other countries [1,6,7] the rate is sometimes even lower: in France, for example, the Hepatitis B vaccine was administered to only 40.1% of children of equal age [8], even though the vaccine against measles, mumps and rubella at 24 months was given to 93%.

The data for Bologna in 2002 show a general rise in the level of coverage for recommended vaccinations in comparison to previous years, especially for anti-Hib. However, even though an increase can be seen in the levels of anti-measles and rubella and the situation in Bologna is on average better than that reported by the rest of the country, the target coverage of 95% envisaged by the National Plan for the Elimination of Measles and Rubella has not yet been achieved. The objectives set by the Plan, to be reached by 2007, are:

- to reduce and maintain the incidence of Congenital Rubella Syndrome (CRS) at levels below 1 case in every 100.000 live-borns.

- to achieve and maintain the elimination of measles at a national level, interrupting its indigenous transmission.

It should be stressed that the inadequate vaccinal coverage achieved for anti-measles does not interrupt the circulation of the virus and may even bring about a greater probability of complications and outbreaks; it prolongs the interval between epidemics and delays the outbreaks until later in adult life, as presumably occurred in the Campania region [9]. It has also been shown that low coverage rates tend to enhance epidemics associated with imported viruses [10]. In 2002 there was quite a large outbreak of measles in Italy. The geographic distribution of cases strictly coincided with that of the vaccine coverage, which is lower in southern Italy [11].

As far as the data on non-compliance (compulsory vaccinations) is concerned, it is interesting to note that the frequency is higher in Bologna than the regional average and, in particular, Bologna East has a higher percentage than Bologna West both at 12 and 24 months. The percentage of total non-compliance is also greater in the East district than the West. Furthermore, the number of children in this category is steadily increasing: passing from 0.88% in 1998 (regional mean 0.40%) to 2.52% in 2002 (regional mean 1.06%).

The most common reason for non compliance is objection of parents. Objection to compulsory vaccinations, especially in Bologna (but not throughout the whole of Italy), is on the rise, even though vaccinal coverage remains above 95%. There is an increasing tendency not to comply with vaccinal obligations, probably because the disappearance (diphtheria, polio) or limited incidence (tetanus, hepatitis B) of certain diseases has led some people to believe that the relative vaccines are obsolete and there is less concern about the risk of contracting infectious diseases. However, recent reports [12] indicate that Italy could risk the importation of the wild polio virus on account of the continuous exchanges with North African countries which are currently in danger of an epidemic due to their negative behaviour towards the vaccination, thus allowing the virus to pass into otherwise polio-free areas.

A further reason for the refusal of vaccination is the anxiety about the possible risks associated with receiving the vaccines. In other words there is the pressure, especially in industrialised countries, from organised groups opposing the practice of vaccination and spreading alarmist and often inaccurate information [13,14]. For example, there is absolutely no scientific evidence that the presence of thiomerosal in vaccines can be linked to the increase in autism [15] or that there is any link between autism and the anti-measles vaccination [16,17]. If the tendency not to vaccinate children becomes more pronounced over the coming years, it could result in serious consequences for public and individual health. It should be remembered that many children still die from preventable infectious diseases and connected pathologies not only in the so-called "poor" countries, but also in other nations. In Europe, the political, social and economic changes that took place following the fall of the Berlin Wall have led to a decline in the levels of immunization in many regions of Eastern and Central Europe, resulting in thousands of new cases of diseases such as diphtheria [18]. For this reason the level of dissent needs to be constantly monitored. The computerized system adopted in Bologna can, in fact, help improve the identification of non-vaccinated subjects and verify the phenomenon of immigration.

## Conclusion

In Bologna the frequency of children aged 12 and 24 months who have achieved compulsory vaccination varied, in 2002, between 95% and 98%; As regards recommended vaccinations the percentage of coverage against Hib is 93.3%, while the levels for measles, mumps and pertussis range from 84% to approx. 92%. Although these percentages are higher if compared to those obtained by other Italian regions, it is necessary to take into account that in Italy, during the 1998–2002 period the mean number of cases of measles, rubella and mumps was 5456, 3685 and 24663 respectively [19]. Therefore, every effort should be made to strengthen the aspects that lead to a successful vaccinal strategy: the continuous improvement in the quality of services, the provision of accurate information to parents and, finally, the training of health workers – in accordance with the suggestions of the WHO – to monitor adverse reactions and maintain an open and constant dialogue with the media about the problems of vaccine safety [18,20].

## Competing interests

The author(s) declare that they have no competing interests.

## Authors' contributions

SS participated in the design and coordination of the study, in the interpretation of results, in revising and editing the manuscript. RR participated in the conception of the study, in the interpretation of the results and in writing the first draft. IR collected the data and performed statistical analyses. FZ participated in the interpretation of the results, the writing of the manuscript and assisted with the statistical analyses and surveys

## Pre-publication history

The pre-publication history for this paper can be accessed here:



## References

[B1] Murray CJL, Shengelia B, Gupta N, Moussavi S, Tandou A, Thierren M (2003). Validity of reported vaccination coverage in 45 Countries. Lancet.

[B2] Salmaso S, Rota MC, Ciofi degli Atti M, Tozzi AE, Kreidl P, e ICONA Study Group (1999). Infant immunization coverage in Italy by cluster survey estimates. Bull World Health Organ.

[B3] Ministry of Health, General Management for Prevention, Office III, Infectious Diseases (2004). Vaccination Coverage in Italy. http://www.ministerosalute.it/promozione/malattie/malattie.jsp.

[B4] DPR 23 luglio 1998 Public Health and Hygiene Approval of the National Health Plan for the three-year period 1998–2000.

[B5] Angelillo IF, Ricciardi G, Rossi P, Pantisano P, Langiano E, Pavia M (1999). Mothers and vaccination: knowledge, attitudes and behaviour in Italy. Bull World Health Organ.

[B6] Antona D, Bussière E, Guignon N, Badeyan G, Lèvy-Bruhl D (2003). Vaccine coverage of pre-school age children in France in 2000. Eurosurveillance Monthly Arch.

[B7] Swennen B, Van Damme P, Vellinga A, Coppieters Y, Depoorter AM (2002). Analysis of factors influencing vaccine uptake: perspectives from Belgium. Vaccine.

[B8] Gaudelus J, Ovetchkine P, Cheimol J, De Courson F, Allaert FA (2003). Suivi des recommendations vaccinales des nourrissans de 0 à 24 mois: è propos d'une enquete en medicine libérale. Arch Pediatr.

[B9] CDC (2003). Measles epidemic attributed to inadequate vaccination coverage, Campania, Italy, 2002. MMWR Weekly.

[B10] Coughlans S., Connel J, Cohen B, Jin L, Hall WW (2002). Suboptimal Measles-Mumps-Rubella vaccination coverage facilitates an imported measles outbreak in Ireland. Clin Infect Dis.

[B11] Echarlod E, Marcoz G, Sudano L (2004). Vaccine coverage and measles outbreak in the Valle d'Aosta: the reality and criticality of a small region. Il Giornale della Vaccinazione.

[B12] Fara GM, Patti AM (2004). What strategies in an Italy without Polio?. Journal of Prev Med Hyg.

[B13] Committee on Community Health Services and Committee on Practice and Ambulatory Medicine (2003). Increasing immunization coverage. Pediatrics.

[B14] Leask J, McIntyre (2003). Public opponents of vaccination: a case study. Vaccine.

[B15] Verstraeten T, Davis RL, De Stefano F, Lieu TA, Rhodes PH, Black SB, Shienfield H, Chen RT, for the vaccine safety Datalink Team (2003). Safety of Thimerosal-containing vaccines. A two phase study of computerized health maintenance organization database. Pediatrics.

[B16] Chen W, Landau S, Sham P, Fombonne E (2004). No evidence for links between autism, MMR and measles Virus. Psychol Med.

[B17] Smeeth L, Cook C, Fombonne E, Heavey L, Rodrigues LC, Smith PG, Hall AJ (2004). MMR vaccination and pervasive developmental disorders: a case-control study. Lancet.

[B18] Panà A (2003). Current situation of vaccines and immunization of the population. Ig Sanita Pubb.

[B19] Carreri V (2004). The commitment of the regions concerning diseases preventable with vaccines. Il Giornale della Vaccinazione.

[B20] Jacobson RM (2003). Vaccine Safety. Immunol Allergy Clin North Am.

